# We Need Studies of the Mortality Effect of Vitamin A Supplementation, Not Surveys of Vitamin A Deficiency

**DOI:** 10.3390/nu9030280

**Published:** 2017-03-15

**Authors:** Christine Stabell Benn

**Affiliations:** 1 Research Center for Vitamins and Vaccines, Bandim Health Project, Statens Serum Institut, Artillerivej 5, 2300 Copenhagen S, Denmark; cb@ssi.dk; Tel.: +45-3268-8354; 2 Odense Patient data Explorative Network, Odense University Hospital/Institute of Clinical Research, University of Southern Denmark, 5000 Odense C, Denmark

**Keywords:** vitamin A deficiency, vitamin A supplementation, child mortality, low-income countries

## Abstract

It is usually acknowledged that high-dose vitamin A supplementation (VAS) provides no sustained improvement in vitamin A status, and that the effect of VAS on mortality is more likely linked to its immunomodulating effects. Nonetheless, it is widely assumed that we can deduce something about the need for continuing or stopping VAS programs based on studies of the biochemical prevalence of vitamin A deficiency (VAD). This is no longer a tenable assumption. The justification for using VAS is to reduce child mortality, but there is now doubt that VAS has any effect on overall child mortality. What we need now are not surveys of VAD, but proper randomized trials to evaluate whether VAS has beneficial effects on overall child survival.

Studies from the 1980s and early 1990s showed that vitamin A deficiency (VAD) was associated with increased overall child mortality [1] and high-dose vitamin A supplementation (VAS) reduced overall mortality [2]. This has led to the long-lived and strong assumption that VAS works by preventing VAD. Though intuitive, this assumption is contradicted by several facts.

First, high-dose VAS has no sustained effect on VAD, as measured by serum retinol or other biochemical markers. Frequent intakes of vitamin A in physiological doses—e.g., through food-based approaches, including fortification, and through regular low-dose supplementation—are highly effective in increasing serum retinol and reducing VAD [3]. However, when the dose of vitamin A is as high as 200,000 IU (about 100 times the daily allowance), the liver may not be able to store it, and the excess is broken up and excreted [4]. Thus, the rise in serum retinol resulting from 6-monthly VAS is small, transient, and lasts only for 1–3 months [5,6].

Second, if VAS worked by preventing VAD, then one would expect a clear linear association between the degree of underlying VAD and the effect of VAS: the higher the prevalence of VAD in a community, the larger the effect of VAS. However, this is not the case. Already, the first meta-analysis of the initial eight studies of the mortality effect of VAS noted that there was no association between the effect of VAS on mortality and the degree of underlying VAD at the population level [2]. As presented in a recent review [7], this conclusion is substantiated when more recent studies are included (Figure 1). 

In their review “Vitamin A Supplementation Programs and Country-Level Evidence of Vitamin A Deficiency” [12], the authors acknowledge that VAS is given to reduce mortality, and does not have any sustained impact on VAD: “Due to VA’s influence on immune function, supplementation with a high dose of VA is designed to reduce mortality associated with measles, diarrhea, and other illnesses [] and not to sustainably improve the VA status of populations. A high dose of VA improves VA status for only up to three months in children who have low dietary intake” [12].

Nonetheless, the authors conclude that “It is widely agreed that VAD data are needed to better target VAS programs and to justify scaling up/down VAS programs” and they clearly believe that the results of VAD surveys should directly influence VAS programs: “Properly done surveys yielding new VAD data will likely have programmatic implications. These surveys may identify specific areas where VAD is rare and the VAS program should be scaled back. In other cases, survey data could suggest that VAS programs should be implemented.” [12].

If VAS is given to reduce mortality, and does not work by preventing VAD, then making the prevalence of VAD determine where to implement or scale back VAS does not make sense. 

It is important to set things right for several reasons. 

National surveys of VAD are difficult and expensive to conduct, because they require collection of blood samples for biochemical analyses. Furthermore, the surveys suffer from a number of limitations, of which the most important are the difficulties of taking into account ongoing inflammation (which would exaggerate VAD if not controlled for), and that the results of national surveys may mask sub-national variation. The authors, many of whom have economic interests in conducting VAD surveys, acknowledge these limitations. 

More importantly, the number one priority for global child health is to reduce mortality. With under-five mortality rates of 73/1000 live births in the least developed countries (World Bank, 2015), that job is far from done. Hence, we should invest in activities with a large potential for reducing mortality, and not waste resources on activities with no impact on mortality. 

VAS has been seen as a golden bullet against child mortality, and enormous resources, both in terms of dollars and opportunity costs, have been invested in providing VAS biannually to millions of children every year. As an unfortunate side-effect, VAS programs may have prevented the implementation of potentially effective programs aimed at reducing VAD, such as food-based solutions including fortification, because policy makers believe that VAD is addressed by VAS, or are concerned about excessive vitamin A intake among the children already receiving VAS [3].

New evidence is now indicating that VAS may no longer be a golden bullet against mortality. The two most recent VAS trials found no evidence for a beneficial effect of VAS, despite being conducted in areas with a high prevalence of VAD [10,11]. The results of these new trials are highly different from the results of previous trials, and are compatible with the interpretation that the overall mortality effect of VAS has ceased to be beneficial [11]. Furthermore, several studies suggest that the mortality effect of VAS, being an immunomodulator, is dependent on a number of factors, which also influence the immune system, such as the most recent vaccines given, sex of the child, and season [7]. Particularly, there are worrying suggestions, that the effect of VAS may be negative for females when given together with DTP-containing vaccines. In contrast, VAS may be very beneficial for females when given with the measles vaccine [7]. As the initial VAS studies were conducted in the pre-vaccination era, the roll-out of vaccination programs may partly explain why VAS may no longer be as effective against child mortality as it was in the 1980s and early 1990s.

The authors agree that “The main rationale for implementing VAS programs is to prevent mortality” [12]. Thus, mortality should therefore be our outcome of interest. What we need now are not VAD surveys. The time has come for proper randomized trials of VAS, to study the mortality effect and define subgroups which may benefit from VAS—maybe even in spite of not being deficient—and importantly also define subgroups, which may be harmed by VAS—maybe even in spite of being deficient.

Only with such data can we decide if and where VAS should be scaled up or scaled back. That decision should not be based on the biochemical prevalence of VAD, when everybody, including the authors, acknowledge that VAS does not work by preventing VAD. There is an urgent need to “de-link” VAS and VAD. VAD should be prevented with food-based interventions and fortification. Mortality should be prevented with clever use of VAS, and that can only be obtained through studies, which focus on mortality. 

## Figures and Tables

**Figure 1:**
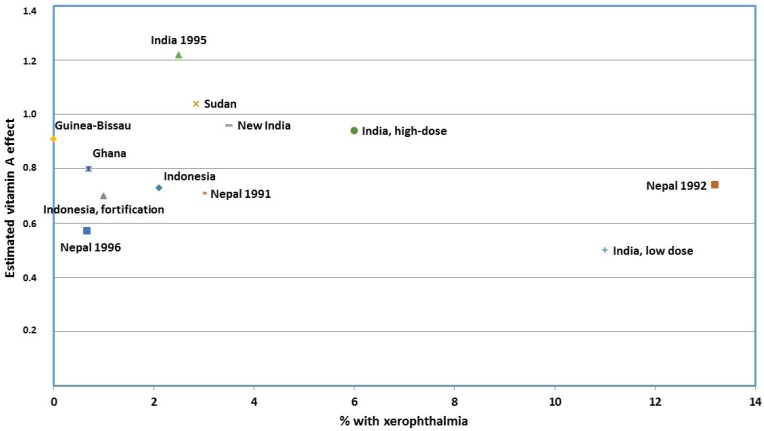
The relative risk comparing vitamin A vs. no vitamin A by prevalence of xerophthalmia in the original eight [2], the two subsequent [8,9], and the two new trials [10,11] of vitamin A supplementation to children above 6 months of age (Modified by Beaton et al., 1993 [2], and first presented in Benn et al., *Int. J. Epidemiol*. 2015 [7]).
